# Ongoing Evolution of Middle East Respiratory Syndrome Coronavirus, Saudi Arabia, 2023–2024

**DOI:** 10.3201/eid3101.241030

**Published:** 2025-01

**Authors:** Ahmed M. Hassan, Barbara Mühlemann, Tagreed L. Al-Subhi, Jordi Rodon, Sherif A. El-Kafrawy, Ziad Memish, Julia Melchert, Tobias Bleicker, Tiina Mauno, Stanley Perlman, Alimuddin Zumla, Terry C. Jones, Marcel A. Müller, Victor M. Corman, Christian Drosten, Esam I. Azhar

**Affiliations:** King Abdulaziz University, Jeddah, Saudi Arabia (A.M. Hassan, T.L. Al-Subhi, S.A. El-Kafrawy, E.I. Azhar); Charité–Universitätsmedizin Berlin, Berlin, Germany (B. Mühlemann, J. Rodon, J. Melchert, T. Bleicker, T. Mauno, T.C. Jones, M.A. Müller, V.M. Corman, C. Drosten); German Center for Infection Research, Berlin (B. Mühlemann, J. Melchert, V.M. Corman, C. Drosten); Ministry of Health and Al-Faisal University, Riyadh, Saudi Arabia (Z. Memish); Emory University, Atlanta, Georgia, USA (Z. Memish); University of Iowa, Iowa City, Iowa, USA (S. Perlman); University College London, London, UK (A. Zumla); University College London Hospitals Biomedical Research Centre, London (A. Zumla); University of Cambridge, Cambridge, UK (T.C. Jones)

**Keywords:** Middle East respiratory syndrome coronavirus, MERS-CoV, coronavirus, coronavirus disease, emerging viruses, virology, respiratory infections, viruses, zoonoses, Saudi Arabia

## Abstract

Middle East respiratory syndrome coronavirus (MERS-CoV) circulates in dromedary camels in the Arabian Peninsula and occasionally causes spillover infections in humans. MERS-CoV diversity is poorly understood because of the lack of sampling during the COVID-19 pandemic. We collected 558 swab samples from dromedary camels in Saudi Arabia during November 2023–January 2024. We found 39% were positive for MERS-CoV RNA by reverse transcription PCR. We sequenced 42 MERS-CoVs and 7 human 229E-related coronaviruses from camel swab samples by using high-throughput sequencing. Sequences from both viruses formed monophyletic clades apical to recently available genomes. MERS-CoV sequences were most similar to B5 lineage sequences and harbored unique genetic features, including novel amino acid polymorphisms in the spike protein. Further characterization will be required to understand their effects. MERS-CoV spillover into humans poses considerable public health concerns. Our findings indicate surveillance and phenotypic studies are needed to identify and monitor MERS-CoV pandemic potential.

Middle East respiratory syndrome coronavirus (MERS-CoV) was first described in 2012 in a human case of viral pneumonia (*1*). Subsequent research uncovered a widespread zoonotic disease caused by a virus that infects humans in the Middle East, East and West Africa, and Pakistan (*2*–*8*). The virus is primarily acquired through direct contact with dromedary camels, its main reservoir host, and with less efficiency through contact with infected humans (*9*–*14*). Infected dromedary camels usually show no or mild clinical signs and quickly recover from infection (*14*). Human-to-human transmission in household and community settings is limited, but nosocomial outbreaks with prolonged interhospital transmission chains have occurred (*15*–*18*). Virus adaptations to humans that cause even subtle changes in transmission probability might lead to an epidemic or pandemic. Because of the zoonotic transmission nature of MERS-CoV, virus evolution in dromedary camel populations is of immediate relevance to humans (*19*).

MERS-CoV is currently classified into clades A, B, and C. Clade A and B viruses are associated with dromedary camels in the Arabian Peninsula; clade C viruses are associated with camels in Africa. Clade A viruses have not been detected since 2015 and might be extinct. Clade B viruses were circulating and evolving in dromedary camels in the Arabian Peninsula at least until 2019 (*20*) and were originally classified into 5 phylogenetic lineages (*21*). Lineage B5 was first reported in 2016, resulting from recombination between lineages B3 and B4. The B5 lineage dominated circulation in Saudi Arabia within 6 months after detection of the first sequence in 2014, and circulation was observed until 2019 (*21*,*22*). Clade C viruses appear to have lower infectivity and virulence (*23*), whereas B5 viruses show increased virulence and fitness in both dromedary camels and humans compared with other A, B, and C clade viruses; those data are from experiments with human epithelial cell and lung explant models (*23*,*24*), hDPP4 transgenic mice (*23*), and camelid models (*25*,*26*).

In addition to MERS-CoV, dromedary camels also harbor a coronavirus closely related to seasonal human coronavirus (HCoV) 229E (subgenus *Duvinacovirus*) (*21**,**27*), highlighting the importance of dromedary camels as a reservoir host for coronaviruses. Because of a lack of sampling during the COVID-19 pandemic, limited knowledge exists regarding the diversity of circulating MERS-CoVs in the Arabian Peninsula (*28*). It remains unknown whether the MERS-CoV lineage B5 continues to dominate in camel populations as it did during 2017–2019 and whether currently circulating MERS-CoVs have polymorphisms that might affect transmissibility or virulence. During January–May 2024, a total of 4 laboratory-confirmed MERS-CoV cases were reported to the World Health Organization by the Ministry of Health of Saudi Arabia (*29*), indicating continuous zoonotic spillover into the human population. Continued surveillance is needed to monitor ongoing changes in MERS-CoV genomes. We report the genetic characterization of 42 MERS-CoV genomes isolated from infected dromedary camels sampled in Saudi Arabia during late November 2023 through early January 2024.

## Methods

### Sample Collection

We collected samples from camels after obtaining ethics approval from the Unit of Biomedical Ethics, King Abdulaziz University Hospital, Rihadh, Saudi Arabia. We collected 572 nasal swabs samples from 576 camels at local camel farms in Jeddah (western Saudi Arabia) and Al Quwaiiyah, Shaqra, Sajir, Al Duwadimi, and Al Riyadh (all locations in central Saudi Arabia). We immersed each swab sample in virus transport medium, transported the samples in a cold container, and stored them at −80°C until further analysis. 

### RNA Extraction and PCR Screening

We extracted virus RNA from 200 μL of sample by using QIAamp Viral RNA Kits (QIAGEN, https://www.qiagen.com) according to the manufacturer’s instructions. We used *upE* and *ORF1A* quantitative reverse transcription PCR to test for MERS-CoV, as described previously (*30*). We considered samples to be MERS-CoV positive if they were PCR positive for both gene targets and had cycle threshold values of <40 (*30*).

### Sequencing

We generated complete genome sequences by using Illumina (https://www.illumina.com) shotgun high-throughput sequencing on selected positive samples and performed subsequent targeted enrichment when necessary. We deposited sequences from this study into GenBank (accession nos. PP952203–9 and PP952162–202). We prepared libraries by using the KAPA RNA HyperPrep Kit (Roche, https://www.roche.com) according to the manufacturer’s instructions. In brief, we fragmented 5 µL RNA at 85°C for 6 minutes. We measured indexed DNA libraries by using the Qubit dsDNA HS Assay Kit (Thermo Fisher Scientific, https://thermofisher.com) and High Sensitivity D1000 ScreenTape Assay Kit for TapeStation (Agilent, https://www.agilent.com). We sequenced equimolar pooled libraries by using an Illumina NovaSeq 6000 system (paired ends, 200 cycles).

We applied a targeted enrichment approach by using myBaits hybridization capture kits (Daicel Arbor Biosciences, https://www.arborbiosci.com) for 29 of the originally sequenced MERS-CoV–positive samples (Appendix 1). We designed a capture bait-set using an alignment of 119 virus sequences, including reference sequences for MERS-CoV (n = 20), SARS-CoV (n = 39), and SARS-CoV-2 (n = 1), as well as the endemic HCoVs: OC43 (n = 20), NL63 (n = 15), 229E (n = 10), and HKU1 (n = 5). The final bait-set comprised a total of 38,279 baits with a length of 80 nt and 3-fold tiling density. Among the generated baits, we did not observe BLAST (https://blast.ncbi.nlm.nih.gov) hits for the following genomes: human, *Sus scrofa* wild boar, *Camelus dromedarius* dromedary camel, or *Myotis lucifugus* little brown bat. We performed targeted enrichment by following the manufacturer’s recommendations. We performed hybridization for 18 hours at 65°C and washing steps at 65°C. We amplified the enriched libraries for 14 cycles by using the KAPA Hifi HotStart Ready Mix and KAPA Library Amplification Primer Mix (both Roche). We sequenced equimolar pooled, purified and quantified libraries on an Illumina MiniSeq instrument (paired ends, 150 cycles, Illumina).

### Bioinformatic Analyses

We trimmed next-generation sequencing reads by using AdapterRemoval v2.3.2 (*31*) and the qualitymax 41, trimns, minlength 30, trimqualities, and minquality 2 options. We mapped reads by using Kraken 2 (*32*) and inspected the resulting krona plots for evidence of infection with HCoV-229E. We mapped reads against MERS-CoV (GenBank accession no. OL622036.1) and HCoV-229E–related CoV (accession no. KT253327.1) sequences by using Bowtie 2 version 2.4.2 (*33*) and the no-unal and local options; we reported only the best match for each read. We merged the binary alignment map files from the native and capture sequencing data by using samtools (https://github.com/samtools/samtools). We called consensus sequences by using iVar v1.3.1 (*34*) and the -m 5 and -t 0.6 options.

We screened all samples for minor variants (https://github.com/VirologyCharite/minor-variants). We assumed a position to be a minor variant if the frequency of the most common nucleotide at that position was <80% and if the position was covered by >5 reads. 

For phylogenetic analysis, we downloaded all available MERS-CoV genomes (GenBank accession no. txid1335626) as of February 29, 2024, by using the search query: txid1335626[Organism:exp]. We reconstructed trees by using IQ-TREE (Appendix 1) (*35*). We filtered sequences to include those that had >29,900 nt and >90% coverage and excluded sequences from bats or those with low quality, leading to a total of 620 genomes. We aligned the sequences by using MAFFT v7.471 (*36*) and the auto and addfragments options; we used GenBank sequence NC_019843 as the reference.

We performed recombination analysis by using RDP4 software (*37*) and included sequences generated in this study and lineage B5 sequences. The automated exploratory analysis implemented in RDP4 uses 7 recombination detection algorithms (RDP, GENECONV, Chimaera, MaxChi, BootScan, SiScan, and 3Seq). We identified recombination events that involved novel sequences, which we confirmed by inferring maximum-likelihood phylogenetic trees from the minor and major parents. We visually inspected pairwise sequence identity plots for B3, B4, and B5 lineages to determine the presence of a similar recombination event between lineages B3 and B4 that formed B5 lineages. Finally, we compared trees constructed from complete genome sequences and trees made from spike sequences to determine whether additional recombination events occurred. We calculated pairwise sequence identities for sequences, excluding invariant sites, by using a window size of 15 and a step size of 1.

## Results

During the camel breeding period of November 2023–January 2024, we sampled a total of 558 dromedary camels from farms in 6 locations in Saudi Arabia: Jeddah (n = 101), Al Duwadimi (n = 90), Al Quwayiyah (n = 97), Al Riyadh (n = 108), Sajir (n = 94), and Shaqra (n = 68). Camels were 1–5 years of age; 291 (52.2%) were <2 years of age. We found 217/558 (38.9%) camels were positive for MERS-CoV RNA by quantitative reverse transcription PCR: Jeddah, n = 36/101 (35.6%); Al Duwadimi, n = 63/90 (70.0%); Al Quwayiyah, n = 21/97 (21.6%); Al Riyadh, n = 58/108 (53.7%); Sajir: n = 28/94 (29.8%); and Shaqra, n = 11/68 (16.2%) (Table; Appendix 2 Table). We selected 51 samples for nontargeted sequencing on the basis of a MERS-CoV load of >2.10 × 10^6^ genome copies/mL. We subsequently used targeted enrichment of coronaviruses for a subset of 29 samples, resulting in 42 MERS-CoV sequences with >99% genome coverage and >5× coverage depth. MERS-CoV sequences were obtained from samples collected during November 22, 2023–January 5, 2024, and came from each of the 6 sampling sites (Jeddah, n = 6; Al Duwadimi, n = 4; Al Quwayiyah, n = 8; Al Riyadh, n = 8; Sajir, n = 12; and Shaqra, n = 4). Nine of the sequenced MERS-CoV–positive samples were co-infected with an HCoV-229E–related CoV with >10% genome coverage, and 7 near-complete HCoV-229E–related CoV genome sequences with >93% coverage were obtained from Jeddah (n = 5) and Sajir (n = 2) (Table; Appendix 2 Table). We found no differences in MERS-CoV virus loads between male and female camels (p = 0.18 by 2-sample *t*-test) or camel age groups (p = 0.12 by Kruskal-Wallis test). However, MERS-CoV loads were significantly lower when a co-infection with HCoV-229E-related CoV was present (p = 0.005 by 2-sample *t*-test).

**Table:** Characteristics of camels with MERS-CoV–positive swab samples across 6 sampling sites, Saudi Arabia, 2023–2024*

Characteristics	Total no.	Sajir	Al Duwadimi	Al Riyadh	Al Quwayiyah	Shaqra	Jeddah
Total no. camels sampled	558	94	90	108	97	68	101
Swab samples taken	576	97	101	108	98	68	104
Younger camels†	291 (52.2)	50 (53.2)	24 (26.7)	60 (55.6)	48 (49.5)	27 (39.7)	82 (81.2)
MERS-CoV–positive camels	217 (38.9)	28 (29.8)	63 (70.0)	58 (53.7)	21 (21.6)	11 (16.2)	36 (35.6)
MERS-CoV–positive samples	235 (40.8)	31 (32.0)	74 (73.3)	58 (53.7)	22 (22.4)	11 (16.2)	39 (37.5)
MERS-CoV–positive camels†	111 (19.9)	14 (14.9)	17 (18.9)	35 (32.4)	12 (12.4)	5 (7.4)	28 (27.7)
Mean PCR Ct (range)	26.2 (10.0–34.0)	23.4 (13.0–32.0)	27.2 (14.0–32.0)	27.9 (14.0–33.0)	24.4 (15.0–32.0)	16.2 (10.0–25.0)	27.7 (11.0–34.0)
Mean PCR Ct (range)†	26.4 (10.0–34.0)	24.5 (13.0–32.0)	27.1 (14.0–31.0)	27.2 (14.0–33.0)	23.9 (15.0–31.0)	14.0 (10.0–17.0)	28.9 (17.0–34.0)
Sequenced samples	51	13	4	8	8	9	9
MERS-CoV genomes, >90% complete	42	12	4	8	8	4	6
HCoV-229E samples, >10% complete	9	3	1	0	0	0	5
HCoV-229E genomes, >90% complete	7	2	0	0	0	0	5

*Values are no. (%) except as indicated. Ct, cycle threshold; HCoV, human coronavirus; MERS-CoV, Middle East respiratory syndrome coronavirus. †Camels <2 years of age.

**Figure 1:**
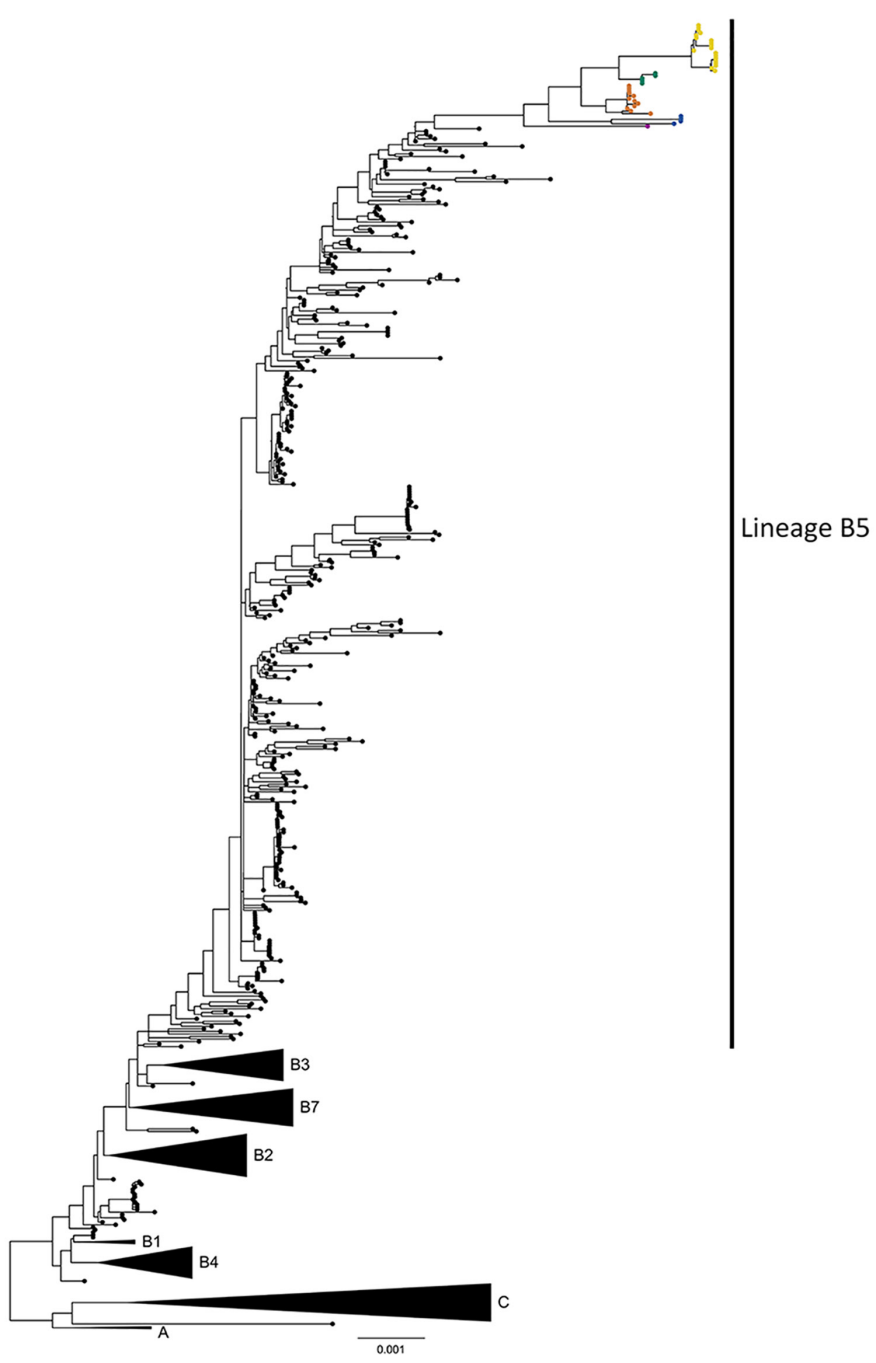
Phylogenetic analysis of Middle East respiratory syndrome coronavirus (MERS-CoV) clades and sample distribution in study of ongoing evolution of virus, Saudi Arabia, 2023–2024. Tree was constructed by using the maximum-likelihood method. Black circles indicate 620 complete MERS-CoV genomes sampled until 2019; colored circles indicate 41 MERS-CoV genomes sequenced in this study. Blue circles indicate B5-2023.1, orange circles B5-2023.2, green circles B5-2023.3, yellow circles B5-2023.4, and magenta B5-2023.5 sublineages. Black triangles indicate collapsed clades A, C, B1–B4, and B7. Scale bar indicates nucleotide substitutions per site.

**Figure 2:**
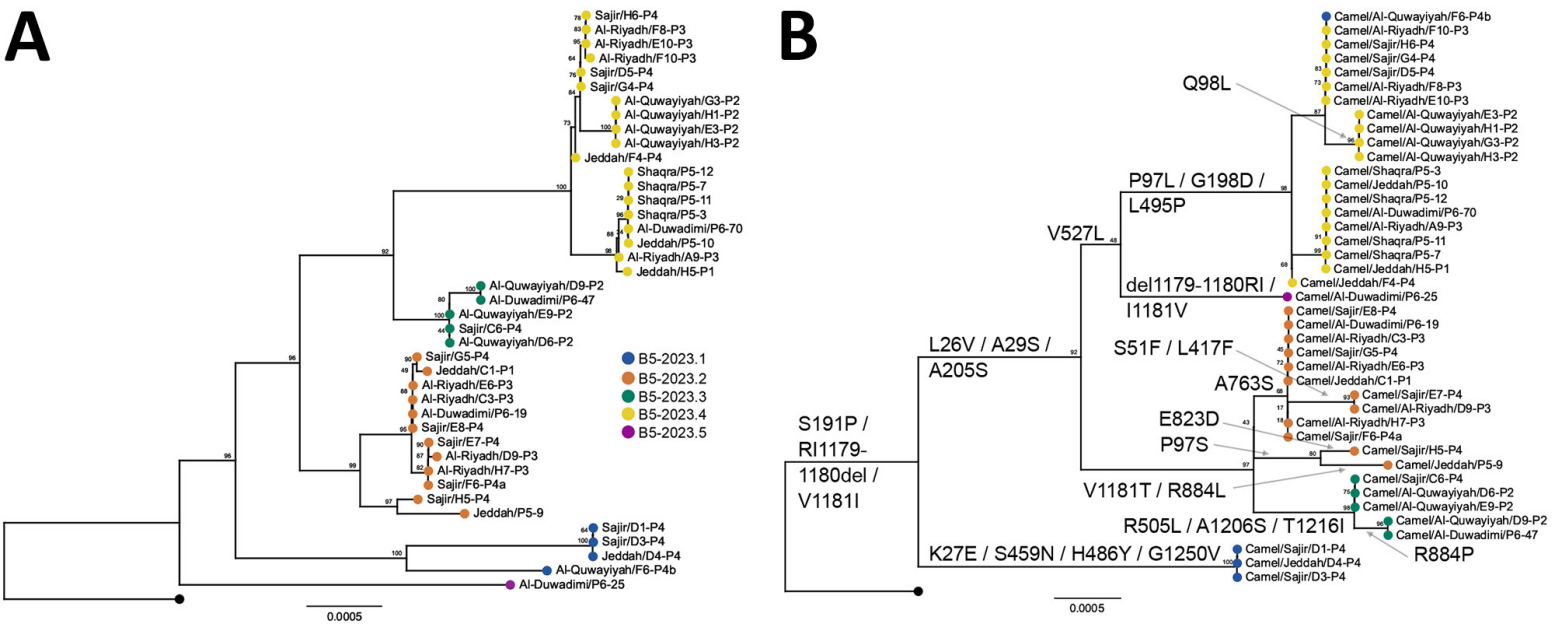
Phylogenetic analyses of Middle East respiratory syndrome coronavirus (MERS-CoV) clade B5-2023 sequences from Saudi Arabia, 2023–2024. Trees were constructed using the maximum-likelihood method. Each tree is rooted with MERS-CoV B5 lineage sequence from 2019 (GenBank accession no. OL622036.1); numbers on nodes indicate bootstrap support. Colored circles indicate B5-2023.1–5 subclades. A) Phylogenetic tree of complete MERS-CoV B5-2023 genomes. B) Phylogenetic tree of spike sequences of MERS-CoV B5-2023 genomes. Amino acid substitutions in the spike protein relative to those of OL622036.1 are indicated on the branches except for substitutions T387P and I743S, which are unique to OL622036.1. The reversion of the R1179–I1180 deletion and V1181I substitution in Al Duwadimi/P6–25 is most likely caused by a recombination event (Appendix 1 Figure 2, panel G). Scale bars indicate nucleotide substitutions per site.

To investigate the potential presence of mixed infections or laboratory contamination of samples, we examined all genomic positions for minor variants (i.e., the majority base was present in <80% of all reads at positions with >5× coverage). The median number of minor variant positions per sample was 1 (range 0–118) (Appendix 1 Figure 1). The sample that had 118 minor variant positions was excluded from further phylogenetic analyses. The remaining MERS-CoV sequences from this study formed a monophyletic clade apical to lineage B5, designated as B5-2023 (Figures 1, 2). The B5-2023 clade is part of an evolutionary pattern of ladder-like phylogenetic topology within lineage B5 (Figure 1). According to a phylogenetic tree inferred from the complete genomes (Figure 1; Figure 2, panel A), 5 sublineages within the B5-2023 clade were differentiated (designated as B5-2023.1–5). 

Recombination analysis of the sequences confirmed a previously described recombination event between lineages B3 and B4 that preceded the formation of lineage B5 (Appendix 1 Figure 2, panels A, B) (*21*). We also found indications of 3 recombination events within the monophyletic B5-2023 clade (Appendix 1 Figure 2). First, sequences from clades B5-2023.1 and B5-2023.4 had breakpoints at positions 17,816 and 29,588, which might have produced the Al Quwayiyah/F6-P4b/B5-2023.1 sequence (Appendix 1 Figure 2, panels C, D). Second, clade B5-2023.3 might have arisen from a recombination event between clades B5-2023.2 and B5-2023.4 through breakpoints at positions 751 and 15,585 (Appendix 1 Figure 2, panels E, F). Third, phylogenetic trees (Appendix 1 Figure 2, panels D, F) suggested that the Al Duwadimi/P6-25/B5-2023.5 sequence might have arisen from a recombination event between a lineage B5 sequence basal to the B5-2023 cluster and clade B5-2023.3 (Appendix 1 Figure 2, panel G). 

**Figure 3:**
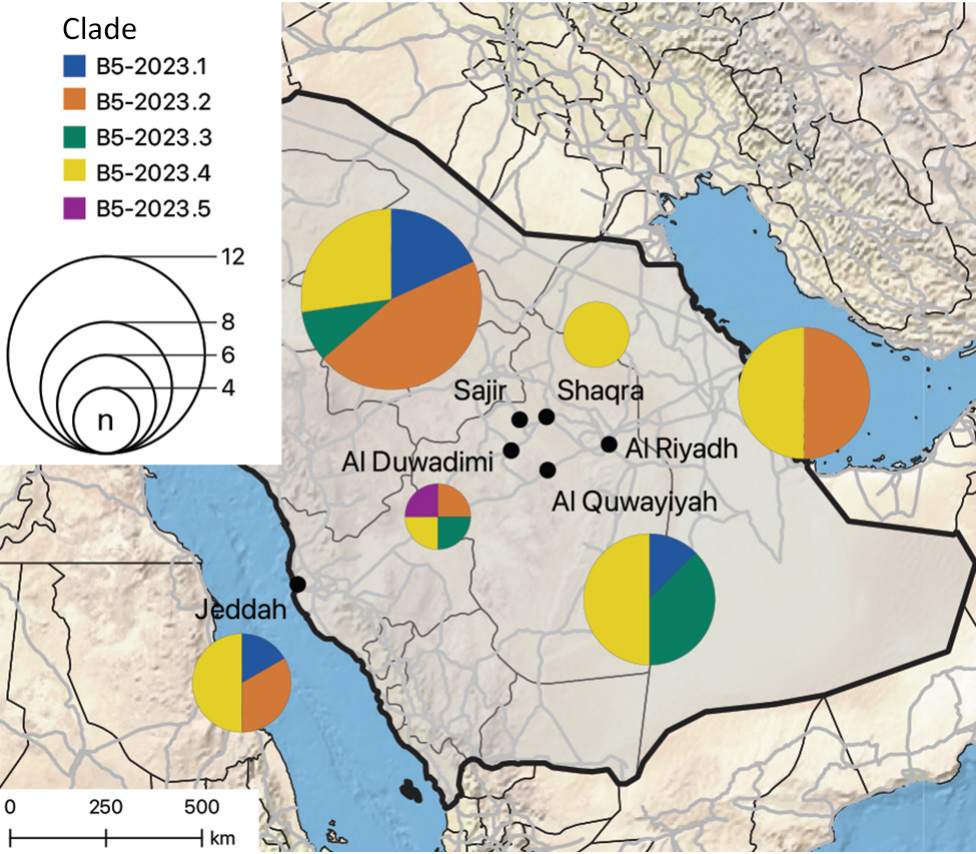
Spatial distribution of B5-2023.1–5 subclades in study of ongoing evolution of Middle East respiratory syndrome coronavirus, Saudi Arabia, 2023–2024. Pie charts show the number of sequences from each subclade found at each of the 6 sampling sites (indicated by black circles on the map) in Saudi Arabia; size of the pie chart corresponds to the number of sequences. Thin black lines indicate administrative regions; gray lines indicate roads. Map was generated by using QGIS v3.28 (https://www.qgis.org).

Regression analyses of root-to-tip distances against sampling dates suggested a constant clock rate across the tree (Appendix 1 Figure 3, panels A, B). The B5-2023 clade acquired 57 polymorphisms compared with the most closely related sequence OL622036.1, which was detected in Saudi Arabia in 2019 (Appendix 1 Table). Those polymorphisms include 2 aa substitutions (S191P and V1181I) in the N-terminal domain and a 2 amino acid deletion (R1179del and I1180del) in the S2 domain of the spike protein. In addition, among the different B5-2023 clade sequences, we observed 23 aa substitutions in the spike protein (Figure 2, panel B; Appendix 1 Table), including in the receptor-binding domain (RBD) (S459N, H486Y, L495P, R505L, and V527L) and in the cathepsin L cleavage site (A763S) (*38*). The sequences did not have deletions in accessory open reading frames, which is often found in clade C viruses (*23*,*39*,*40*). We did not find geographic clustering of clade B5-2023 sublineages; 5 of the 6 sampling sites showed circulation of >2 sublineages, and all but 1 sublineage were detected in >3 of the 6 sampling sites (Figure 3).

**Figure 4:**
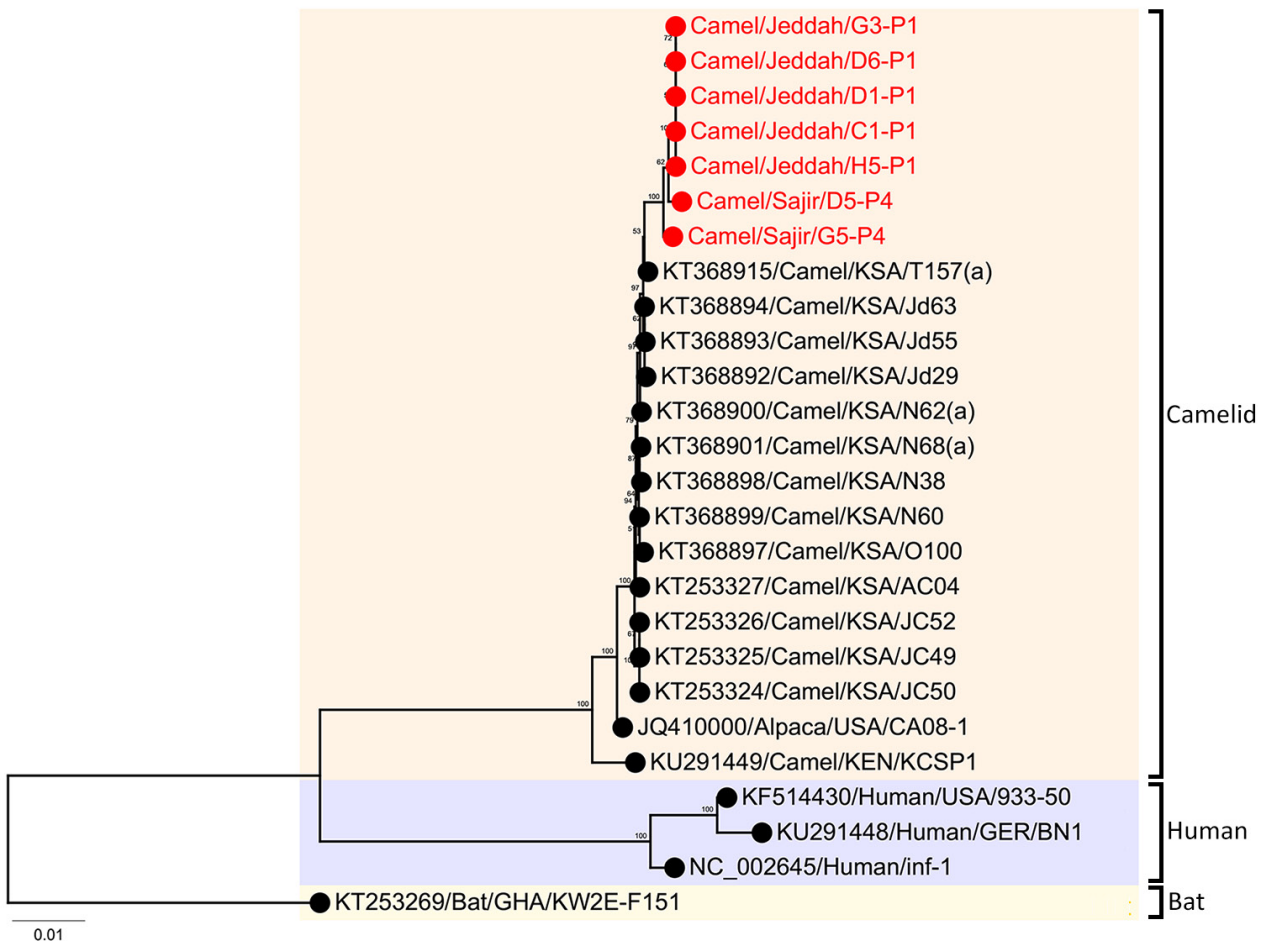
Phylogenetic analysis of 26 human coronavirus 229E-related coronavirus sequences in study of ongoing evolution of Middle East respiratory syndrome coronavirus, Saudi Arabia, 2023–2024. Tree was constructed by using the maximum-likelihood method. Red text indicates sequences from this study, 5 from Jeddah and 2 from Sajir. Numbers on nodes indicate bootstrap support. Tree was rooted with the bat sequence KT253269/Bat/GHA/KW2E-F151 (GenBank accession no. KT253269). Scale bar indicates nucleotide substitutions per site. GER, Germany; GHA, Ghana; KEN, Kenya; KSA, Kingdom of Saudi Arabia; USA, United States of America.

The 7 HCoV-229E–related CoV sequences formed a monophyletic clade apical to previously detected HCoVs from dromedary camels sampled in 2014–2015 (Figure 4). Regression of root-to-tip distances and sampling dates (Appendix 1 Figure 3, panel C) showed a constant clock rate for those data. No deletions in open reading frame 8 (*27*) were found.

## Discussion

We identified a monophyletic clade of MERS-CoV, designated as B5-2023, circulating in Saudi Arabia during 2023–2024. Although previously circulating clade B lineages arose from deep splits within the phylogenetic tree, the apical branching and ladder-like tree topology of the B5-2023 sequences suggest that MERS-CoV strains circulating in the Arabian Peninsula during 2023–2024 originated solely from the B5 lineage. This supposition is also supported by recombination analysis and evolutionary indications of a constant molecular clock rate shown in this study.

We identified 25 aa substitutions and 2 aa deletions in the spike protein among the different MERS-CoV B5-2023 clade sublineages (Figure 2, panel B), including in the RBD and N-terminal domain (Appendix 1 Table). The RBD is the site most frequently targeted by neutralizing antibodies from humans infected with MERS-CoV (*41*). Two of the substitutions found in the B5-2023 clade (L495P and V527L) are situated on the ridge of the receptor-binding motif exposed in the closed conformation of the spike protein, an epitope preferentially targeted by antibodies found in human serum samples after MERS-CoV infection (*41*,*42*). Furthermore, a substitution in the cathepsin L cleavage site (A763S) found in clade B5-2023.3 might affect spike protein cleavage and virus infection in cells that do not express transmembrane protease, serine 2 (*38*). Those amino acid substitutions require further study to determine their effects on virus entry, receptor affinity, immune escape, and replicative fitness.

The distinct sublineages in clade B5-2023 did not cluster geographically, indicating that dromedary camels are maintaining virus diversity across different sites within the central Arabian Peninsula. The reservoir traits of dromedary camels include rapid virus clearance, waning adaptive immune responses, and evidence of rapid reinfection (*43*–*47*); thus, it is likely that parallel evolution of distinct MERS-CoV sublineages is ongoing in dromedary camels. This concept is consistent with studies on MERS-CoV genetic diversity conducted before 2020 (*21*,*48*) and consistent with the detection of 3 recombination events within the B5-2023 clade. The movement of camels for grazing and leisure promotes mixing of populations from different regions (*49*), which might enhance MERS-CoV spread across the Arabian Peninsula.

Since the beginning of the COVID-19 pandemic, few human cases of MERS-CoV have been reported in Saudi Arabia, in stark contrast to the large epidemic outbreaks reported during 2012–2019. The small number of reported MERS-CoV infections might be because of limited MERS-CoV surveillance and nonpharmaceutical interventions that were in place during the COVID-19 pandemic or because of phenotypic changes in circulating MERS-CoV. We are unable to speculate on the cause of the reduced number of reported human cases of MERS-CoV. However, our findings highlight the urgent need for in-depth epidemiologic and spatiotemporal studies to identify hotspots of MERS-CoV dissemination and areas that have high risk for human spillover. Furthermore, phenotypic characterization will be required to better understand the potential for MERS-CoV spread in the human population.

We observed co-infections with HCoV-229E-related CoV and MERS-CoV in 18% of the 51 sequenced samples from camels, similar to previous observations (*21*,*27*,*50*). The apical placement of the newly described HCoV-229E-related CoV sequences together with temporal signal might also point to a ladder-like pattern of evolution for that virus in dromedary camels. Similarities in the epidemiology of HCoV-229E-related CoV and MERS-CoV in dromedary camels, including the absence of severe disease and the higher rate of infection in younger animals (*14*,*27*), suggest that HCoV-229E-related CoV might be maintained at population levels similar to that of MERS-CoV and that dromedary camels are critical reservoir hosts for coronaviruses.

A limitation of our study is that, because the sampling was performed in 6 locations, 5 in central and 1 in western Saudi Arabia, it is possible that other MERS-CoV lineages circulating in different regions of the Arabian Peninsula were not detected. Furthermore, sample collection was performed during a season of typically high MERS-CoV incidence and only camels <6 years of age were sampled.

In conclusion, spillovers of MERS-CoV into the human population in the Arabian Peninsula pose a substantial public health concern, which is highlighted by the enhanced replicative fitness and transmission capabilities of B5 lineage viruses in dromedary camels (*23*–*26*). The ongoing MERS-CoV genetic evolution revealed by the sequencing data in this study highlights the urgent need for further MERS-CoV surveillance and phenotypic studies to monitor MERS-CoV spillover, adaptation, and pandemic potential.

Appendix 1 Additional information for ongoing evolution of Middle East respiratory syndrome coronavirus, Saudi Arabia, 2023–2024.

Appendix 2 Sequencing metadata for all tested swab samples from camels.
