# Supplementary material for: Ongoing Evolution of Middle East Respiratory Syndrome Coronavirus, Saudi Arabia, 2023–2024
Source: Emerg Infect Dis. 2025 Jan;31(1):57–65. doi: 10.3201/eid3101.241030 (PMC11682817; doi:10.3201/eid3101.241030)
Supplement: Appendix 1 — Additional information for ongoing evolution of Middle East respiratory syndrome coronavirus, Saudi Arabia, 2023–2024. [file 24-1030-Techapp-s1.pdf]

Article DOI: https://doi.org/10.3201/eid3101.241030

EID cannot ensure accessibility for supplementary materials supplied by authors. Readers who have difficulty accessing supplementary content should contact the authors for assistance.

Ongoing Evolution of Middle East Respiratory Syndrome Coronavirus, Saudi Arabia, 2023–2024

Appendix 1

Additional Methods

We designed a targeted enrichment approach for sequencing by using myBaits hybridization capture kits (Daicel Arbor Biosciences, https://www.arborbiosci.com) for 29 of the originally sequenced Middle East respiratory syndrome coronavirus (MERS-CoV)–positive samples. We designed a capture bait-set using an alignment of 119 virus sequences. The bait-set can be ordered from BioCat GmbH (https://www.biocat.com) using our initial reference number 200408–94. We performed targeted enrichment as previously described for other viruses (*1–3*) by following the manufacturer's recommendations.

Maximum likelihood phylogenetic trees were inferred by using IQ-TREE v2.2.0.3 (*4*). The substitution model was determined by performing an initial run on the full alignments with the options -m MF and -nt AUTO (*5*), which showed the general time reversal (GTR) + empirical base frequencies (F) + free rate with 4 categories (R4) substitution model was optimal for MERS-CoV and human coronavirus (HCoV)-229E alignments. Subsequent IQ-TREE runs were performed by using the -m GTR+F+R4 -B 1000 -nt AUTO options and 1000 ultrafast bootstraps. Regression of root-to-tip distances against sampling dates was performed in TreeTime v0.11.3 (*6*). The trees from the previous IQ-TREE runs were used as input for the full MERS-CoV and HCoV-229E trees. To investigate the temporal signal in lineage B5, a separate tree was inferred as described by using just MERS-CoV lineage B5 sequences and the B5-2023 sequences described in this study. Figures were plotted in Python v3.10 by using the matplotlib

(*7*), seaborn (*8*), and baltic (https://github.com/evogytis/baltic) packages. The map was generated by using QGIS v3.28 (https://www.qgis.org).

References

1. Ruscher C, Patzina-Mehling C, Melchert J, Graff SL, McFarland SE, Hieke C, et al. Ecological and clinical evidence of the establishment of West Nile virus in a large urban area in Europe, Berlin, Germany, 2021 to 2022. Euro Surveill. 2023;28:2300258.

2. Schilling-Loeffler K, Viera-Segura O, Corman VM, Schneider J, Gadicherla AK, Schotte U, et al. Cell culture isolation and whole-genome characterization of hepatitis E virus strains from wild boars in Germany. Microorganisms. 2021;9:2302.

3. Melchert J, Radbruch H, Hanitsch LG, Baylis SA, Beheim-Schwarzbach J, Bleicker T, et al. Whole-genome sequencing reveals insights into hepatitis E virus genome diversity, and virus compartmentalization in chronic hepatitis E. J Clin Virol. 2023;168:105583.

4. Nguyen LT, Schmidt HA, von Haeseler A, Minh BQ. IQ-TREE: a fast and effective stochastic algorithm for estimating maximum-likelihood phylogenies. Mol Biol Evol. 2015;32:268–74.

5. Kalyaanamoorthy S, Minh BQ, Wong TKF, von Haeseler A, Jermiin LS. ModelFinder: fast model selection for accurate phylogenetic estimates. Nat Methods. 2017;14:587–9.

6. Sagulenko P, Puller V, Neher RA. TreeTime: maximum-likelihood phylodynamic analysis. Virus Evol. 2018;4:vex042.

7. Hunter JD. Matplotlib: a 2D graphics environment. Comput Sci Eng. 2007;9:90–5.

8. Waskom M. seaborn: statistical data visualization. J Open Source Softw. 2021;6:3021.

9. Sabir JSM, Lam TTY, Ahmed MMM, Li L, Shen Y, Abo-Aba SEM, et al. Co-circulation of three camel coronavirus species and recombination of MERS-CoVs in Saudi Arabia. Science. 2016;351:81–4.

Appendix 1 Table. Clade-defining amino acid and nucleotide substitutions in Middle East respiratory syndrome coronavirus B5-2023 lineage and B5-2023.1–5 sublineages compared with the closest B5 lineage sequence OL622036.1*

Sequence	B5-2023†	B5-2023.1‡	B5-2023.2	B5-2023.3	B5-2023.4§	B5-2023.5
Protein						
ORF1ab	S378P, E532A, T642I, L759F, D1174E, G1767D, S1841L, A1977T, C2131G, R2932K, D3889E, A4473D, F6442V, S6494A	S611N, N682K, V698A, T782I, V1034I, N1147S, T1740M, I2265T, S5305A, T6911I	Y1888H, T1933I	None	Q512H, D671N, V5820I, V6749F	S393N, R1573G, S2138N, F2531L, Y2631F, R2961K, L4623F, M5086I, L5777F
S	S191P, T387P, I743S, RI1179–1180del, V1181I	K27E, S459N, H486Y, G1250V	None	R505L, A1206S, T1216I	P97L, G198D, L495P	None
ORF3a	C38F, I44V, L86F	P7S	None	None	None	S101L
ORF4a	None	None	None	None	None	None
ORF4b	None	None	None	None	None	L41F, K151N
ORF5	I147T, V191A	None	None	None	None	None
E	None	None	None	None	None	None
M	None	None	None	None	None	None
N	N22H	K92R, D126N, S171L, S198G	None	None	None	A5S
ORF8b	None	*S27G*, H106Y	None	None	None	P4L, Q68L
Genome	*T701C,* A1322G, C1361T, T1374C, A1873C, C2167T, T2243C, C2517T, T3764G, *C4160T,* T4955C, *G5542A, C5764T,* G6171A, T6633G, G6899A, *T8456C, G9037A, C9152T, T9368G,* T9441G, T11213C, T11747G, C11828T, C11909A, T12056A, T13456C, C13659A, C16327T, T16618C, C16765T, C16843T, T17164C, A18934C, G19396A, T19565G, T19721G, C20236T, T20857C, T21990C, A22578C, T23647G, *C23852T,* G24163A, C24347T, T24791G, *24957–24962del,* G25608T, A25625G, T25695C, C25751T, T26641C, T27243C, T27375C, C28117T, C28221T, A28593C	C126T, G506T, T764C, T947C, G2074A, T2288G, T2335C, C2587T, G3342A, C3383T, A3682G, C4739T, G6959A, T7036C, G7058A, T7847C, 8479–8481del, C10265T, C11630T, C13184T, T14867C, T16154G, T16189C, T16870C, *C20973T*, *A21082G, T21154C, A21498G, G22391T, G22795A, G22865A, C22875T, C23474T, C23942A, T25034C, G25168T*, *C25514T*, *C26452T, T26782C, C27889T, G28668T, A28840G, G28905A, C29041T, A29121G, T29166C, T29330C*	C128T, T761C, T3042C, C5126T, C5516T, T5904C, C6040T, C6743T, C9620T, C12323T	G112A, G12484T, G22933T, G25035T, C25066T, T27769C	G1778T, G2253A, G6206A, G16879T, G17699A, *A17863T*, *C17923T, C18490T, C18913T, T19277T, G20486T, T21019C, C21709T, G22012A*, *T22903C, T25334G*	C251T, G1420A, C3470T, T4637C, A4959G, C5912T, G6655A, G6770T, T7779C, A8134T, T8138C, A9124G, C9704T, A9947G, A9998G, C10049T, C12119T, G12482A, T13732C, C14108T, C15421T, G15499T, C16441T, C16846T, C17570T, T18298C, G20305A, C20563A, T23036C, C23588T, T24884C, T25561C, C25797T, C25890T, C26013T, T26161C, C26177T, G26230A, G26509T, C26611T, C26896T, C28374T, G28542T, G28682A, C28738T, A28928T, T29405C

*Closest B5 lineage sequence from 2019 (GenBank accession no. OL622036.1). Substitutions in B5-2023.1–5 sublineages only include substitutions unique to each sublineage. E, envelope; M, membrane; N, nucleocapsid; ORF, open reading frame; S, spike.
†Substitutions in italics are not present in the possible recombinant B5-2023.5 sublineage (Appendix 1, Figure 2, panel G).
‡Substitutions in italics are not present in the recombinant sequence Al Quwayiyah/F6-P4b. Sequence 'Al Quwayiyah/F6-P4b' is a recombinant between a sequence from sub-lineage B5–2023.1 and B5–2023.4 (Appendix 1, Figure 2, panels C, D).
§Substitutions in italics are also present in sequence Al Quwayiyah/F6-P4b.

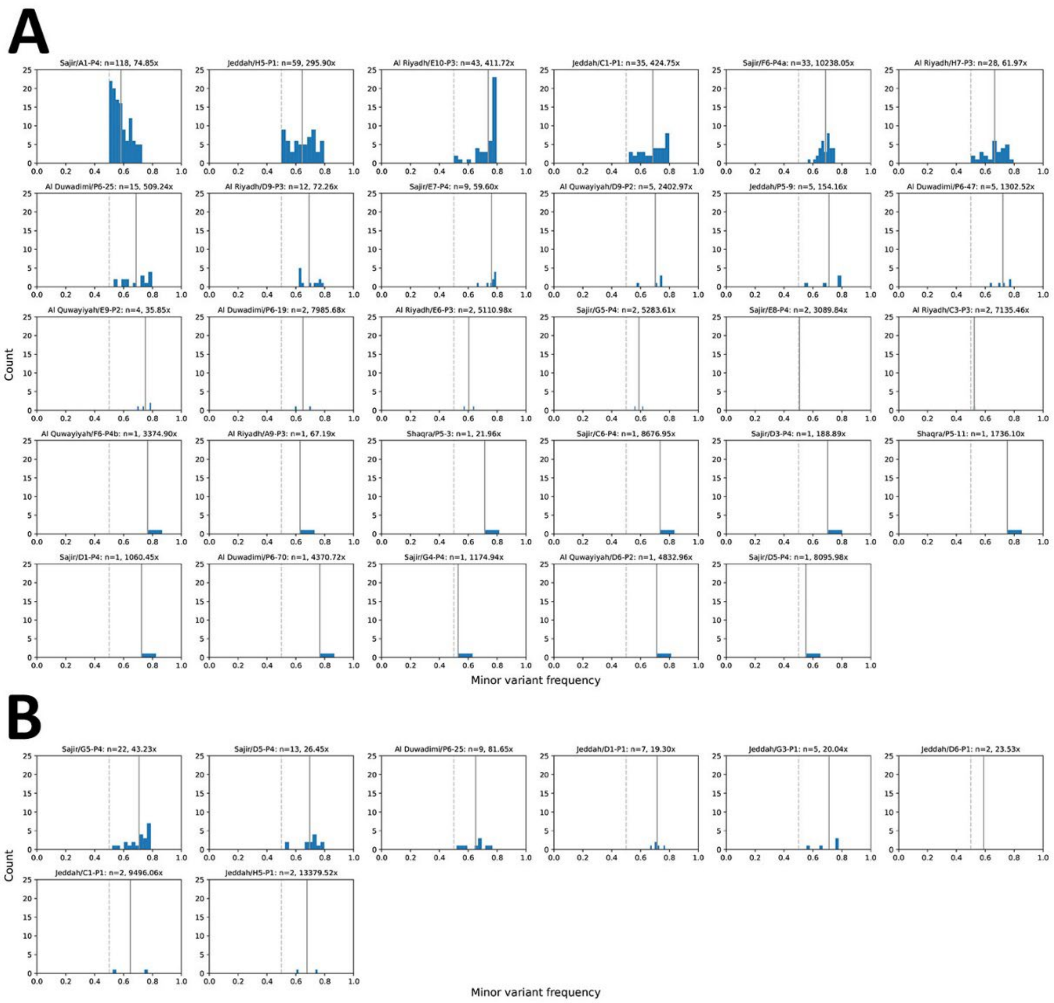

Appendix 1 Figure 1. Frequency distribution of the most common base at minor variant sites in study of Middle East respiratory syndrome coronavirus, Saudi Arabia, 2023–2024. Frequencies are indicated for Middle East respiratory syndrome coronavirus (A) and human coronavirus-229E–related coronavirus (B). A site is defined as a minor variant site if it is covered by >5 reads and the frequency of the most common base is <0.8. Solid vertical lines indicate the mean; dashed lines indicate 0.5 minor variant frequency. Title of each plot indicates the sample name, number of minor variant sites, and mean coverage depth of the entire sample in that order.

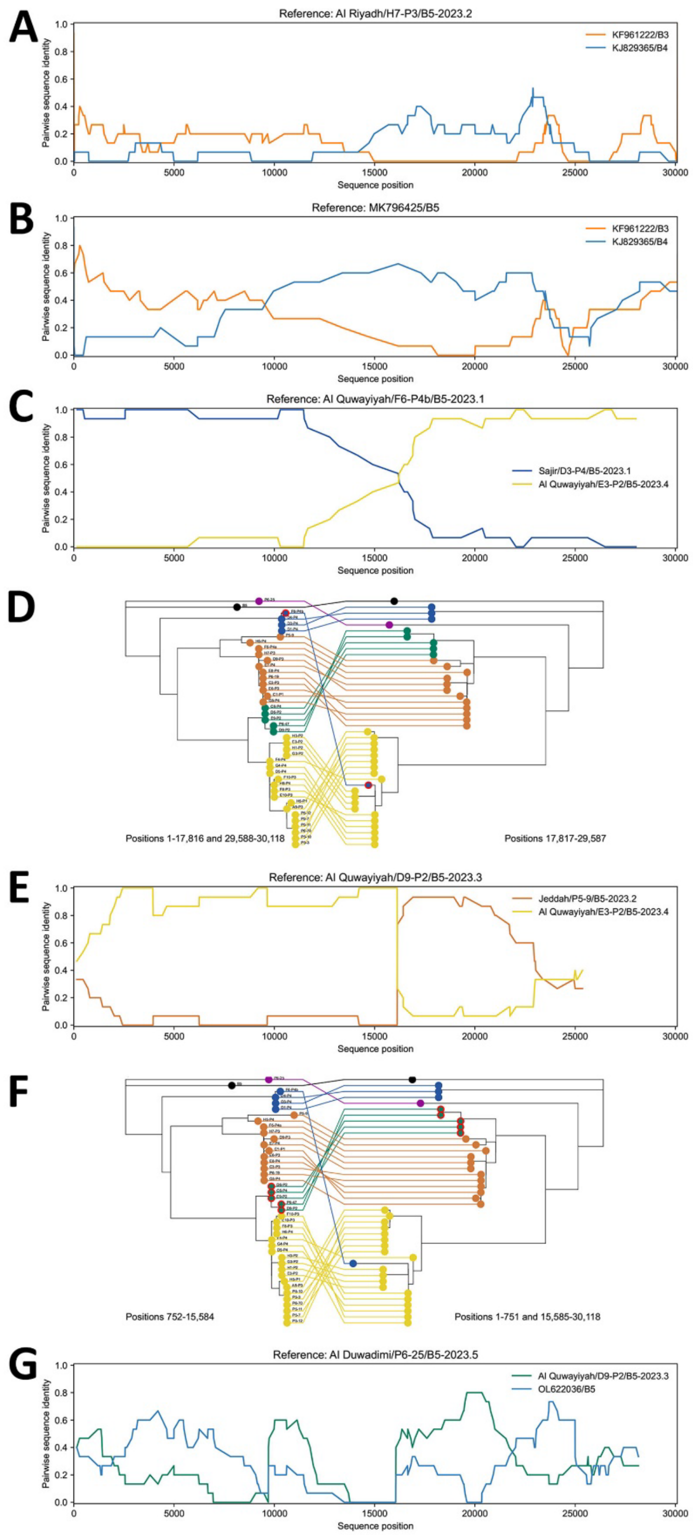

Appendix 1 Figure 2. Recombination analysis of clade B Middle East respiratory syndrome coronavirus. A, B) Plots showing a previously described recombination event between lineages B3, B4, and B5 (*9*). Pairwise sequence identity plots for lineages B3, B4, and B5-2023 (Al Riyadh/H7-P3/B5-2023.2) (A), and

B3, B4, and a B5 sequence (GenBank accession no. MK796425) (B). C, D) Sequence Al Quwayiyah/F6-P4b/B5-2023.1 from this study results from a recombination event between B5-2023.1 subclade (blue) and B5-2023.4 subclade (yellow). C) Pairwise sequence identity plot between sequences from Al Quwayiyah/F6-P4b/B5-2023.1 and B5-2023.1 (Sajir/D1-P4/B5-2023.1) or B5-2023.4 (Al Quwayiyah/E3-P2/B5-2023.4) subclades. D) Trees made from genomic sequences of the major (major: 1–17,816 and 29,588–30,118) and minor (offsets: 17817–29,587) parents. Blue circles indicate B5-2023.1, orange circles B5-2023.2, green circles B5-2023.3, yellow circles B5-2023.4, and magenta B5-2023.5 sublineages; red outlines indicate recombinant sequences. Tree was rooted with OL622036.1 (black circles). E, F) Sequences in the B5-2023.3 clade arose from a recombination event between B5-2023.2 and B5-2023.4 subclades. E) Pairwise sequence identity plot between sequences from B5-2023.3 (Al Quwayiyah/D9-P2/B5-2023.3) and B5-2023.2 (Jeddah/P5-9/B5-2023.2) or B5-2023.4 (Al Quwayiyah/E3-P2/B5-2023.4) subclades. F) Trees made from genomic sequences of the major (offsets: 1–751 and 15,585–30,118) and minor (offsets: 752–15,584) parents. Colored circles are described in (D). G) Pairwise sequence identity plot indicating a possible recombination event, producing Al Duwadimi/P6-25/B5-2023.5, between a sequence from clade B5–2023.3 (Al Quwayiyah/D9-P2/B5-2023.3) and an older B5 lineage sequence, represented here by OL622036/B5. Pairwise sequence identities shown in panels A–C and E were calculated by excluding invariant sites and by using a window size of 15 and a step size of 1. Panels D and F show trees inferred from the parents; lines connect the same sequence in the 2 trees.

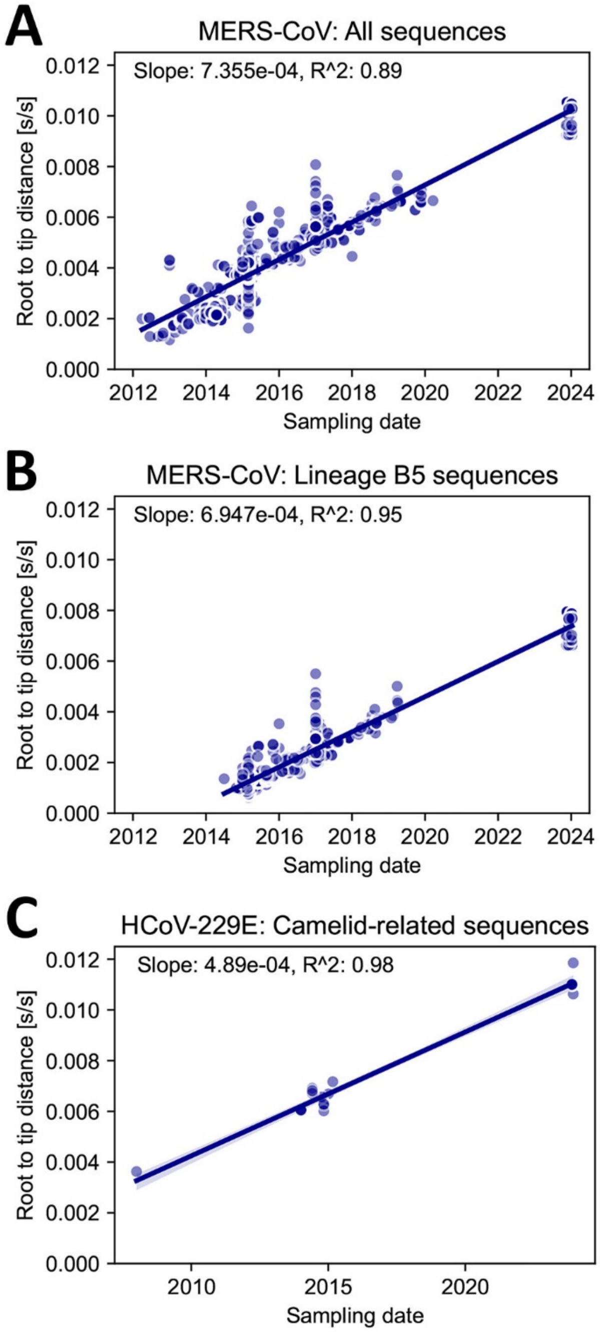

Appendix 1 Figure 3. Regression analysis of root-to-tip distances versus sampling dates for Middle East respiratory syndrome coronavirus (CoV) and related CoVs. Root-to-tip distances are plotted against sampling dates for the full phylogenetic tree (Figure 1, panel A, main text) (A), for lineage B5 and the new clade B5-2023 (B), and for the human CoV-229E–related CoV sequences (C). Sampling dates that had unknown months or days were set to the first of the month or January 1 of the year the sequence was sampled. Lines indicate least-squares regression.
